# Size-Resolved PM_10_-Bound PFASs: Respiratory Deposition and Association with Oxidative Stress Biomarkers Among Industrial Waste Recycling Workers

**DOI:** 10.3390/toxics14080682

**Published:** 2026-08-03

**Authors:** Xing Li, Lei Zheng

**Affiliations:** 1 School of Public Health, Zhengzhou University, Zhengzhou 450001, China; 2 Guangdong Key Laboratory of Environmental Pollution and Health, College of Environment and Climate, Jinan University, Guangzhou 510632, China; 3 Beijing SDL Technology Co., Ltd., Beijing 102206, China

**Keywords:** oxidative stress, per- and polyfluoroalkyl substances, size-resolved airborne particulate matter, internal and external exposure, exposure–response analysis

## Abstract

The global surge in industrial waste generation has raised substantial concerns regarding occupational exposure to per- and polyfluoroalkyl substances (PFASs) and the associated adverse health effects. However, critical knowledge gaps remain regarding the relationships among size-resolved airborne particulate matter (PM)-bound PFASs, internal exposure, and early-stage health damage. We conducted a panel study at a waste recycling plant in southern China, collecting 15 size-resolved PM samples and 280 repeated first morning void urine samples from 20 workers over 45 consecutive days. Relationship between size-resolved PM-bound PFASs, internal exposure, and oxidative stress biomarkers (OSBs:8-hydroxy-2′-deoxyguanosine (8-OHdG), malondialdehyde (MDA)) were analyzed using linear mixed-effects models. Our results indicate that short-chain PFASs predominated in both matrices. Intraclass correlation coefficients (ICCs) for urinary short-chain PFASs ranged from fair to excellent (ICC > 0.4). Notably, inhalation of PFASs in PM_2.1–10_ correlate more strongly with internal exposure than those in PM_2.1_. For the first time, we observed suggestive quantitative associations between OSBs and multiple short-chain PFASs in urine. More importantly, 8-OHdG levels were mainly correlated with PFASs in PM_4.7–5.8_ and PM_9.0–10_, with each unit increase in ln-transformed average daily intake (ADI) associated with a 10.2–12.6% increase in urinary 8-OHdG levels (*p* < 0.05). Similarly, MDA levels were primarily associated with PFASs bound to PM_5.8–9.0_, with each unit increase in ln-transformed ADI corresponding to an 8.0–14.6% increase in MDA (*p* < 0.05). These findings are exploratory and warrant further confirmation in independent cohorts. Nevertheless, this study highlights the need for more exposure monitoring and health impact assessments regarding PM_2.1–10_-bound short-chain PFASs among the occupational population.

## 1. Introduction

Per- and polyfluoroalkyl substances (PFASs) are a class of synthetic organic compounds widely used in consumer and industrial applications due to their unique water- and oil-repellent properties [1,2]. Their environmental persistence and mobility have resulted in widespread contamination of air, water, soil, and food systems [3]. Human exposure to PFASs has been associated with a range of adverse health effects, including cardiovascular disease [4], neurological impairments [5] and an increased risk of cancer [6]. Micro-exposed people refer to those who are not directly engaged in PFAS manufacturing but are exposed to substantial quantities of PFAS-containing products during their work [7]. Health concerns regarding a large number of micro-exposed population are receiving increasing attention. For instance, significantly higher urinary PFAS concentrations and increased cancer risk have been reported in e-waste recycling workers and firefighters [8,9]. China is the world’s largest producer of industrial waste, and other major consumer countries export substantial quantities of recyclable waste to China and other Southeast Asian regions. Consequently, the risks posed by ubiquitous PFASs exposure in such micro-exposed population warrant urgent investigation.

Airborne particulate matter (PM) is recognized as a major environmental reservoir for toxic pollutants [10,11,12], and PFASs have been detected in both indoor and outdoor dust as well as airborne PM across multiple countries [13,14,15,16]. Particle size critically influences respiratory deposition of PM and subsequent health outcomes [17,18,19]; however, the extent to which size fractions affect inhalation exposure and subsequent health effects of PM-bound PFASs remains poorly understood [20,21].

Urine and serum are the primary biological matrices used to assess internal exposure to PFASs; however, they reflect different exposure windows and compound-specific profiles. Short-chain PFASs have relatively short biological half-lives (days to months) and are efficiently eliminated via renal excretion, making them readily detectable in urine [22,23,24]. In contrast, long-chain PFASs (e.g., PFOS, PFOA) have much longer half-lives (months to years), exhibit strong protein-binding affinity, and accumulate in serum [7,25]. Thus, serum is suitable for assessing long-term exposure to long-chain PFASs, whereas urine provides a noninvasive matrix for capturing recent exposure to short-chain analogs [7,26,27]. Importantly, the temporal variability of internal PFASs exposure remains poorly characterized, and reliance on single spot samples in large-scale studies may lead to exposure misclassification [28,29].

Oxidative stress is a well-established mechanism through which environmental pollutants exert toxic effects [20,21]. Oxidative stress biomarkers (OSBs), such as 8-hydroxy-2′-deoxyguanosine (8-OHdG; a marker of DNA damage) and malondialdehyde (MDA), are indicative of lipid peroxidation and serve as early molecular-level indicators of biological impairment [30,31]. Epidemiological studies have linked various environmental pollutants to oxidative stress and related downstream diseases [32,33,34,35]. Moreover, exposure to long-chain PFASs in serum or plasma has been associated with elevated OSB levels in diverse populations [36,37,38]. However, despite the increasing use of short-chain PFASs as substitutes, studies examining their associations with early biological effects in micro-exposed people remain limited. Furthermore, no study has yet investigated the association between inhalation exposure to size-resolved PM-bound PFASs and OSBs. Beyond conventional PM components, it remains unclear whether PFASs act as key contributors to the adverse health outcomes associated with airborne PM.

To address these gaps, we conducted a panel study at a waste recycling plant in 2022, collecting 15 size-resolved indoor PM samples and 280 first morning void urine samples from 20 workers over a 45-day consecutive period. By integrating repeated size-resolved PM measurements, longitudinal urinary biomonitoring, and a physiologically based deposition model, this study offers a novel framework to link inhalation exposure, internal exposure, and early health impairment at a molecular level. The research content of this study includes (1) characterizing the size distribution and congener profiles of airborne PM-bound PFASs, (2) estimating the average daily inhalation dose (ADI) of size-resolved PM-bound PFASs and evaluating potential noncarcinogenic risks, (3) characterizing the congener profiles and temporal variability of urinary PFASs, (4) investigating the relationship between size-resolved inhalation ADI and corresponding urinary PFASs concentrations, and (5) examining associations between PFASs exposure (external and internal) and variations in urinary OSBs. The primary objective is to identify quantitative relationships among size-resolved PM-bound PFASs, internal exposure levels and oxidative stress biomarkers (OSBs) and elucidate how particle size drives the transition from external exposure to internal dose and early health damage.

## 2. Materials and Methods

### 2.1. Study Population and Data Collection

This study was conducted at a large-scale waste recycling plant in Panyu District, Guangzhou, Southern China, which processes approximately 20,000 tons of metal and plastic waste daily. From August to October 2022, size-resolved airborne PM was collected inside the main processing warehouse using an Anderson eight-stage cascade impactor (TISCH-Model TE-20-800, Cleves, OH, USA) at a flow rate of 28.3 L/min. The impactor aerodynamic cut points are 0.43, 0.65, 1.1, 2.1, 3.3, 4.7, 5.8, and 9.0 μm. The monitoring period was divided into fifteen 3-day cycles. PM samples were collected onto pre-combusted quartz fiber filters (Ø 81 mm, Whatman QMA, Marlborough, MA, USA) every three consecutive workdays (7:30 a.m. to 8:00 p.m. each day) within each cycle, and a total of 15 integrated PM samples were eventually collected. Concurrently, meteorological parameters, including temperature, relative humidity, wind speed, and air pressure, were recorded.

In total, 20 participants (10 males and 10 females) were recruited, their demographic characteristics are shown in Table 1. During the PM monitoring period, first morning void urine samples were collected from each worker on one of the three days. A total of 280 urine samples were obtained (expected: 300; missing: 20) and immediately transported to the laboratory, where they were stored at −80 °C until analysis. This study primarily focuses on short-chain PFASs. Because short-chain PFASs have relatively short biological half-lives (hours to days), same-cycle matching was used without incorporating additional lag structures. All participants provided written informed consent and completed a questionnaire to collect demographic information (age, sex), occupational history (job role, duration of exposure in the warehouse), lifestyle factors (dietary habits), and health metrics including body mass index (BMI), blood pressure, and serum lipid levels. This study was approved by the Ethics Review Board of Jinan University (No. JNUKY-2021-0222) and was conducted in accordance with the Declaration of Helsinki. Written informed consent was obtained from all participants.

### 2.2. Chemicals and Reagents

Details of the 26 targeted PFASs, along with 8-OHdG, MDA, and other chemicals and reagents, are provided in the Supplementary Materials.

### 2.3. Determination of PFASs in Size-Resolved PM and Urine

PM-bound PFASs and urinary PFASs were extracted according to previously established methods with minor modifications [7,39,40]. Briefly, one-quarter of each filter and 2 mL of urine were spiked respectively with 1 ng of a mixed solution of ten internal standards (20 μL, 50 ng/mL), and extracted three times with methyl tert-butyl ether (MTBE). The combined extract was then concentrated under a gentle nitrogen stream and fixed with methanol to 300 μL.

Quantitative analysis of the 26 target PFASs was performed using an ultra-performance liquid chromatography system (Nexera-XZ UPLC, Shimadzu, Kyoto, Japan) coupled to a triple quadrupole mass spectrometer (AB SCIEX Triple Quad 6500, Foster City, CA, USA) in negative electrospray ionization mode. Detailed information regarding sample preparation, extraction procedures and LC-MS/MS parameters is provided in the Supporting Information (Supplementary Materials, Table S1).

### 2.4. Determination of Urinary 8-OHdG and MDA

Urinary creatinine and 8-OHdG were prepared using a direct dilution method [41]. Urinary MDA was derivatized following the previous method [42].

All three analytes were quantified using the LC-MS/MS described above operating in positive electrospray ionization mode. Chromatographic separation was achieved on a Betasil C18 column (100 mm × 2.1 mm i.d., 5 μm particle size; Thermo Electron, Waltham, MA, USA). Detailed procedures for sample preparation and LC-MS/MS analysis have been described in our previous publication [18].

### 2.5. Quality Assurance/Quality Control (QA/QC)

Before sampling, quartz fiber filters were pre-baked at 450 °C for 4 h to remove organic contaminants. All laboratory utensils and containers were rinsed three times with methanol to minimize background contamination. To avoid PFASs adsorption onto glass surfaces, only polypropylene materials were used throughout sample preparation and analysis.

For each batch of samples, two procedural blanks, two blank quartz filters, two spiked blanks, and two spiked matrix samples (randomly selected from the batch) were processed concurrently to assess background contamination, filter interference, and matrix effects. During instrumental analysis, two procedural methanol blanks and two standard mixture references were injected after every 20 samples to monitor carryover and ensure instrument stability.

For PM samples, mean recoveries of target analytes ranged from 70 ± 6% to 83 ± 6% in spiked blanks and from 72 ± 6% to 84 ± 6% in spiked matrices. Recoveries of internal standards ranged from 72 ± 8% to 90 ± 8% (Supplementary Materials, Table S4). For urine samples, analyte recoveries ranged from 68 ± 4% to 73 ± 6% in spiked blanks and from 59 ± 3% to 88 ± 2% in spiked matrices, and internal standard recoveries ranged from 69 ± 4% to 103 ± 7% (Supplementary Materials, Table S5).

Calibration curves exhibited excellent linearity (r^2^ > 0.99) for all target analytes. PFAS concentrations detected in blank quartz filters and procedural blanks were subtracted from those in field PM samples and urine specimens. The limit of detection (LOD) was defined as the lowest concentration on the calibration curve corresponding to a signal-to-noise ratio of 3 (Supplementary Materials, Tables S4 and S5).

### 2.6. Exposure Model and Statistical Analysis

#### 2.6.1. Respiratory Deposition and Health Risk Assessment

Particle size critically influences PM deposition efficiency (DE) in the human respiratory tract. Fine PM tend to deposit in deeper regions, such as the pulmonary alveoli, where they may translocate across the alveolar barrier into systemic circulation more easily than coarse PM [43]. Size-resolved PM DE was calculated using the simplified Human Respiratory Tract Deposition Model as recommended by the International Commission on Radiological Protection (ICRP) [44,45]. The deposition flux (DF_lux_) of PM-bound PFASs was estimated based on DE values. The ADI of PM-bound PFASs via inhalation was calculated using the following equation:

$${DF}_{lux}=\sum \left(DE\times C\right)\times R_{\mathrm{inh}},$$

$$ADI=\frac{{DF}_{lux}\times ET\times EF\times ED}{BW\times AT},$$

$$HQ=\frac{DI}{RfD\;or\;TDI},$$

where C is the PFAS concentration in PM (pg/m^3^), R_inh_ is the inhalation rate (m^3^/h) derived from the basal metabolic rate (BMR) (kJ/d). ET is the daily exposure time (h/day), EF is the annual exposure frequency (335 days/year), ED is exposure duration (30 years), BW is body weight (kg), and AT is the averaging period (30 × 365 days/year) [19]. Potential non-carcinogenic risks were evaluated using the hazard quotient (HQ) method, calculated as ADI divided by the reference dose (RfD) or tolerable daily intake (TDI). RfD values for PFOA and PFOS were obtained from the U.S. EPA, while TDI values for PFHxA, PFHpA, PFNA, PFDA, and PFBS were referenced from previous studies [46]. More detailed information about the ICRP and parameter are provided in the Supplementary Materials (Tables S2 and S3).

#### 2.6.2. Statistical Analysis

Statistical analyses were performed using IBM SPSS Statistics 25 software. PFAS congeners with a detection frequency (DF) below 50% in PM or urine were excluded from further statistical analysis to ensure data reliability and focus on prevalent compounds.

To assess the temporal variability of urinary PFAS levels, intraclass correlation coefficients (ICCs) were calculated using two-way random-effects models. ICC represents the proportion of total variance attributable to between-individual differences and was interpreted according to established guidelines: excellent (≥0.75), fair to good (0.40–0.75), or poor (<0.40) [47,48].

Descriptive statistics were used to summarize PFAS concentrations in PM and urine. Normality was assessed using the Shapiro–Wilk test. Given the repeated measures design, general linear models were initially used to compare PFAS exposure levels across multiple groups. Linear mixed-effect (LME) models with participant-specific random intercepts were then applied to analyze the associations between the ADI of PM-bound PFASs and urinary PFASs with OSBs. A compound symmetry (CS) covariance structure was assumed for the repeated measurements because it provided a better model fit, as indicated by a lower Akaike Information Criterion (AIC), than the unstructured or identity covariance structures. Because the LME model can inherently accommodate unbalanced data, missing observations were excluded without imputation. The ADI of PM-bound PFASs, as well as urinary PFASs and OSB concentrations were ln-transformed to approximate normal distributions. A covariate was retained if its inclusion altered the effect estimate for the primary exposure by ≥5%, based on both biological plausibility and statistical considerations [49]. Finally, covariates including sex, age, smoking status, BMI, job role, blood pressure, blood glucose, and blood lipids were considered for multivariable models. The percent change in the outcome associated with each unit increase in the ln-transformed exposure was calculated as 100% × [exp(β) − 1], where β is the regression coefficient from the mixed model. A two-tailed *p*-value < 0.05 was considered statistically significant. Although the number of independent participants was relatively small (n = 20), the repeated measures design yielded 280 observations, thereby enhancing the statistical power of the analysis. The residuals were randomly distributed without discernible trends, and the Q–Q plot points closely followed a straight line, indicating no apparent violations of the model assumptions. Given the novelty of this study and the lack of prior epidemiological evidence regarding the associations between size-resolved PM-bound PFASs and OSBs, we did not apply stringent multiple-comparison corrections (e.g., Bonferroni correction), as doing so could increase the risk of Type II errors and potentially obscure true associations. Therefore, the observed associations should be considered exploratory. We verified the model assumptions using residual diagnostics and Q–Q plots, and employed built-in robustness checks such as covariate adjustment and outlier exclusion. As the results were stable, we did not conduct additional sensitivity analyses.

## 3. Results

### 3.1. Respiratory Deposition and Health Risk of PM-Bound PFASs

**Figure 1:**
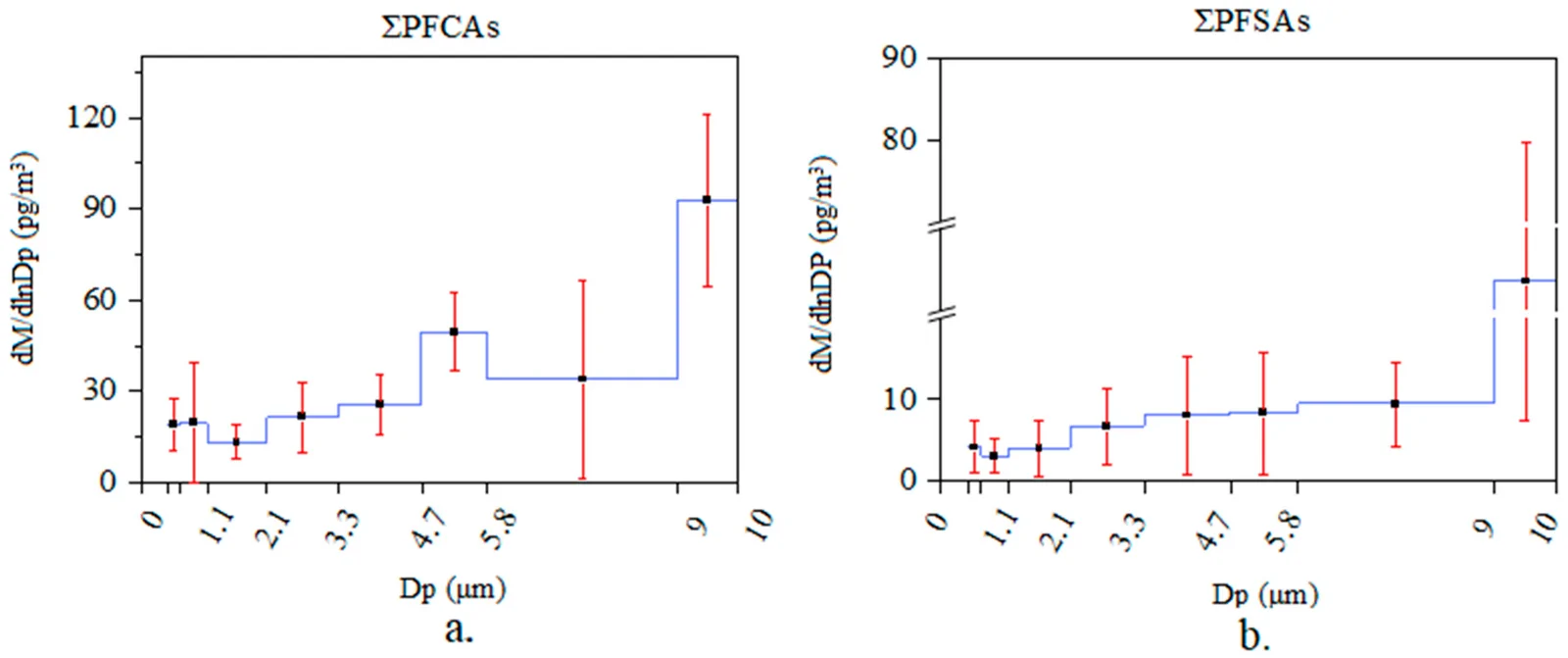
The distribution of average ∑PFCAs (**a**) and ∑PFSAs (pg/m^3^) (**b**) in PM at the waste recycling plant in Southern China. (Red error bars indicate standard deviations (n = 15); Dp represents the aerodynamic diameter (μm)).

Among the 26 targeted PFASs, most short-chain congeners exhibited DFs exceeding 50%. Notably, PFBA (94%), PFOA (92%), and PFHxA (96%) were detected in more than 90% of samples (Supplemental Material, Table S6). The mean daily concentrations of total PFCAs (∑PFCAs: 82.7 ± 21.5 pg/m^3^) were higher than those of total PFSAs (∑PFSAs: 22.6 ± 9.28 pg/m^3^). PFBA (45.8 ± 13.4%) was the dominant contributor to ∑PFCAs, followed by PFPeA (13.5 ± 7.23%), PFOA (12.4 ± 3.89%), and PFHxA (12.2 ± 4.94%). For ∑PFSAs, PFBS (45.1 ± 17.2%) was the predominant congener, followed by PFOS (37.7 ± 15.3%) and PFHxS (17.2 ± 6.64%). The size distributions of ∑PFCAs and ∑PFSAs exhibited trimodal and bimodal patterns, respectively, with peaks at 9.0–10 μm, 4.7–5.8 μm, and 0.65–1.1 μm (Figure 1). Approximately 63% of ∑PFCAs and 70% of ∑PFSAs mass was concentrated in the PM_2.1–10_ size fraction.

**Figure 2:**
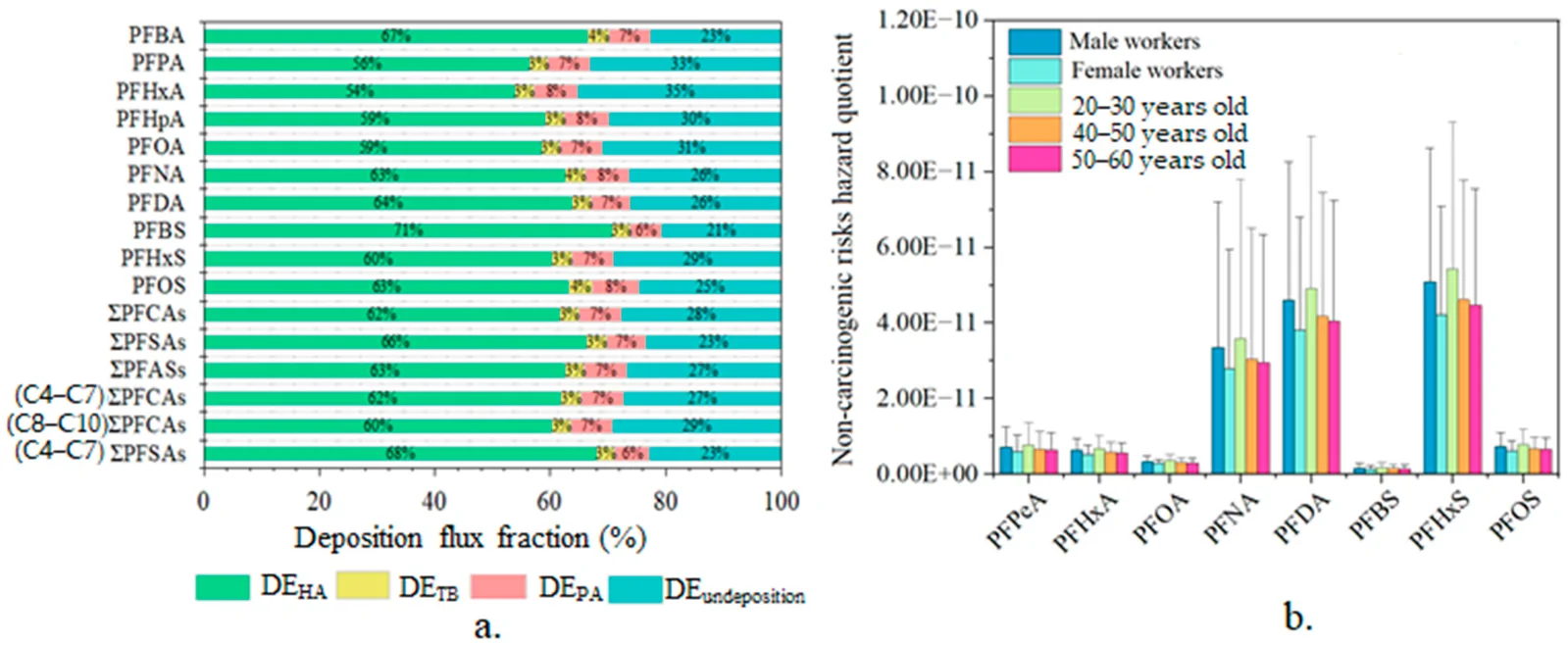
(**a**) Deposition flux fractions of PM-bound PFASs in whole respiratory tract among workers; (**b**) Non-carcinogenic HQ of PM-bound PFASs in whole respiratory tract among workers. Abbreviations: hazard quotients, HQ; head airway, HA; tracheobronchial, TB; and pulmonary, PA. Un-deposition is the fraction of PFASs not deposited in respiratory tract during respiration.

The estimated DF_lux_ of total PFASs (∑PFASs) in the respiratory tract of workers was 44.4 ± 15.9 pg/h, with ∑PFCAs contributing 78% ± 7%. The majority of inhaled PFASs were deposited in the HA, accounting for 54–71% of total deposition (Figure 2a). In contrast, deposition in the TB (3–4%) and PA regions pg/h (6–8%), was substantially lower. Although the relative contribution of PM_2.1_ to ∑PFASs deposition increased with depth in the respiratory tract, PM_2.1–10_ remained the dominant size fraction, accounting for more than 76% of total PFAS deposition (Supplementary Materials, Figure S1). The estimated overall daily intake, derived from pharmacokinetic modeling based on serum concentrations in similar waste recycling workers, was 0.39 and 0.62 ng/kg body weight/day, respectively [7,50]. In contrast, the contribution from inhalation of PM was minimal. The calculated HQ for all target PFASs were significantly below the safety threshold of 1 (Figure 2b), indicating inhalation of PM-bound PFASs in this occupational setting does not pose a significant noncarcinogenic risk to workers. Nevertheless, the HQ analysis is a simplified, single-pathway screening tool and does not rule out potential health effects arising from chronic low-dose exposure through multiple pathways or from interactions with other pollutants.

### 3.2. Urinary PFASs Concentrations and Temporal Variability

#### 3.2.1. Demographic Characteristics

Among the 20 participants (10 males and 10 females), the mean age was 46 ± 10 years, and the mean BMI was 24.7 ± 3.4 kg/m^2^ (Table 1). Workers were categorized into three groups based on job titles and daily exposure duration in the warehouse: (1) short-term exposure (truck drivers, n = 4, 1 h/day), long-term exposure (sorters, stevedores, n = 12, 10 h/day), and indirect exposure (managers, n = 4, 0 h/day). The prevalence of overweight, hypertension, hyperlipidemia and hyperglycemia among workers was 65%, 20%, 35% and 10%, respectively. The prevalence of overweight (73%), hypertension (34%), hyperlipidemia (47%), and hyperglycemia (14%) among directly exposed workers was higher than that in the indirect exposure group (50%, 27%, 34%, and 0%, respectively).

#### 3.2.2. Concentration Profiles and Demographic Associations

Among the 26 targeted PFASs, PFBA (99%), PFPeA (99%), PFHxA (95%), PFOA (99%), PFBS (94%), and PFHxS (99%) were detected in more than 90% of samples (Supplementary Materials, Table S7). The mean urinary concentration of ∑PFASs was 1234 ± 929 ng/L, with ∑PFCAs contributing 80 ± 16% of the total PFASs burden. Among all targeted congeners, PFPeA exhibited the highest mean concentration (825 ng/L), followed by PFHxS (200 ng/L), PFBA (70.5 ng/L), PFOA (69.9 ng/L), and PFBS (23.6 ng/L).

Drivers and loaders exhibited 57–61% and 29–91% higher concentrations of ∑PFCAs and ∑PFSAs, respectively, than office managers. Interestingly, office managers, with long work tenure, showed ∑PFASs concentrations comparable to those of sorters (Table 2). Sex-specific differences were evident, with males exhibiting significantly higher concentrations of both ∑PFSAs (337 vs. 158 ng/L, *p* < 0.01) and ∑PFCAs (1097 vs. 874 ng/L) compared with females (Supplementary Materials, Figure S2). Smoking was associated with an approximately twofold increase in urinary ΣPFSAs concentrations. Overweight individuals (Chinese criteria: BMI ≥ 24) and those with hypertension or hyperlipidemia exhibited higher urinary PFASs concentrations. Notably, among directly exposed workers, the prevalence of overweight (73%), hypertension (34%), hyperlipidemia (47%), and hyperglycemia (14%) exceeded that in the indirect exposure group (50%, 27%, 34%, and 0%, respectively), suggesting a potential association with PFASs exposure that warrants further investigation.

#### 3.2.3. Temporal Variability and Reproducibility of Urinary PFASs

Over the 45-day monitoring period, ICCs for creatinine-adjusted short-chain PFASs, including PFBA, PFPeA, PFHxA, and PFHxS, ranged from fair to excellent in the directly exposed group (Table 3). In contrast, except for PFOA, long-chain congeners exhibited poor reproducibility (ICCs < 0.4). Creatinine adjustment consistently reduced both inter- and intra-individual variability and improved the temporal stability of most short-chain PFASs.

To further characterize the reliability of exposure assessment across varying sampling windows, we calculated the probability of achieving an ICC > 0.40 for individual PFASs over randomly selected consecutive 3n-day periods, using sorters as a representative subgroup. Reproducibility probabilities for PFNS, PFDA, and PFTrDA declined markedly with increasing sampling duration; for example, for PFDA and PFTrDA, probabilities decreased from 67% over 6 days to 0% over 36 days (Supplementary Materials, Figure S3). Conversely, extended sampling intervals markedly improved reproducibility probabilities for PFBS and PFOS. Notably, PFOA and PFHxS consistently achieved 100% reproducibility across all sampling intervals up to 15 days, indicating exceptional temporal stability.

### 3.3. Association Between Size-Resolved PM-Bound PFASs and Urinary PFAS Levels

**Figure 3:**
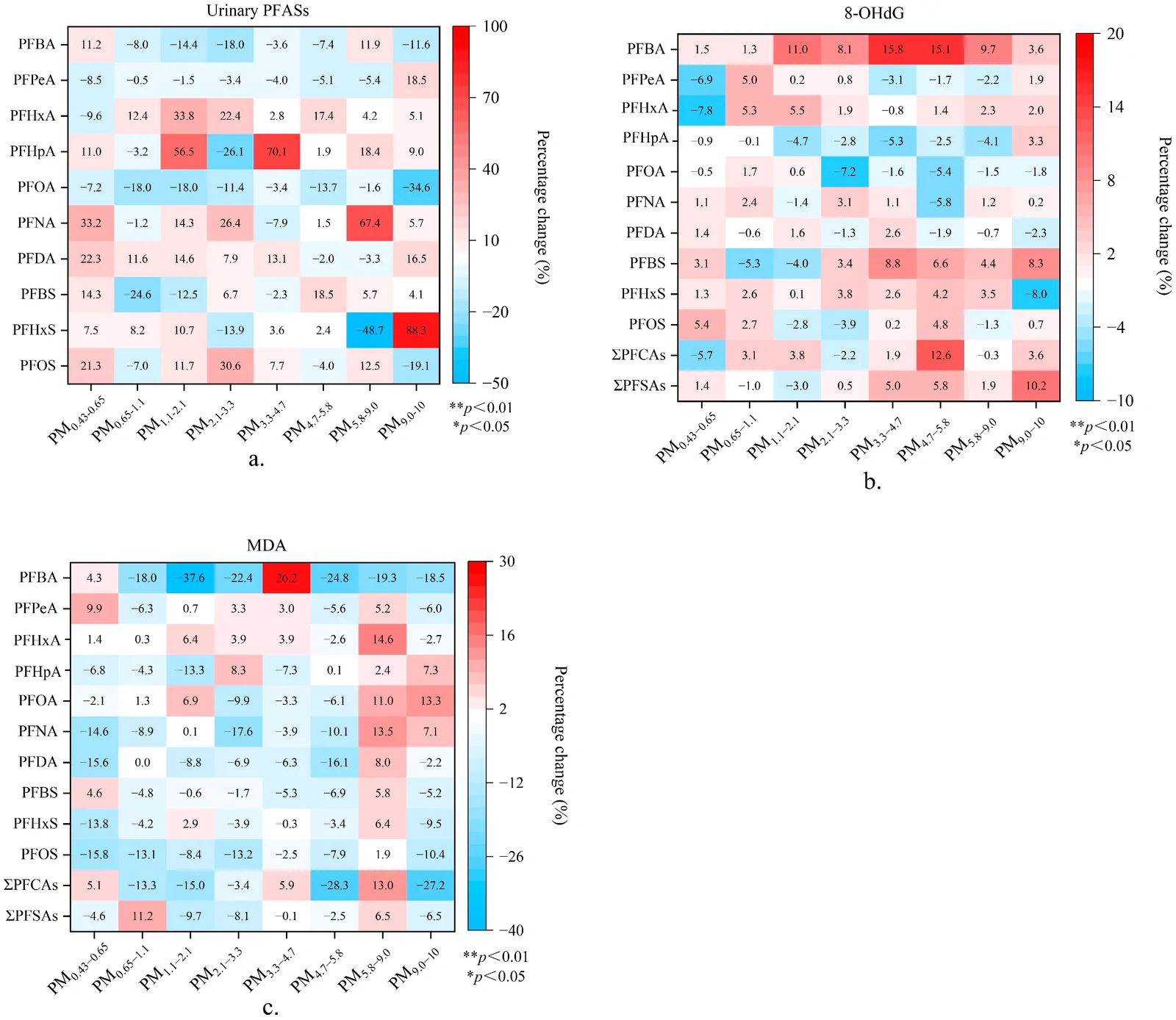
Estimated percentage variations in urinary creatinine-adjusted PFAS levels (**a**), 8-OHdG (**b**), and MDA (**c**) corresponding to each unit rise in ln-transformed of daily intake of size-resolved PM-bound PFASs through inhalation among warehouse employees, as determined by LME models that account for factors such as gender, age, smoking habits, BMI, job roles, length of employment, blood pressure, glucose levels, and lipid profiles. Warehouse personnel include sorters, stevedores, and others engaged in warehouse activities for 10 h. Abbreviations: 8-hydroxydeoxyguanosine, 8-OHdG; malondialdehyde, MDA.

To quantitatively evaluate the contribution of inhaled size-resolved PM-bound PFASs to internal exposure, LME models were applied to examine the associations between ln-transformed urinary PFASs concentrations and ADI of PM-bound PFASs via inhalation in the long-term exposure group. Except for PFBA and PFOA, significant associations were observed between most urinary PFASs and their corresponding airborne concentrations, with the magnitude of these associations varying markedly across particle size fractions (*p* < 0.05; Supplementary Materials, Table S8). For example, urinary PFPeA and PFHxS exhibited the strongest associations with the ln-transformed ADI of PFASs in the PM_9.0–10_ fraction, with each unit increase in ln-ADI corresponding to increases of 18.5% and 88.3% in urinary concentrations, respectively (*p* < 0.05) (Figure 3a). Urinary PFBS concentrations were primarily associated with the PM_4.7–5.8_ fraction (18.5% increase per unit ln-ADI, *p* < 0.01), whereas elevated urinary PFOS concentrations exhibited the strongest association with the PM_2.1–3.3_ fraction (30.6% increase per unit ln-ADI, *p* < 0.01). Overall, associations with ADI derived from coarse particles (PM_2.1–10_) were consistently stronger than those derived from fine particles (PM_2.1_).

### 3.4. Associations Between Urinary PFASs and Oxidative Stress Biomarkers

**Figure 4:**
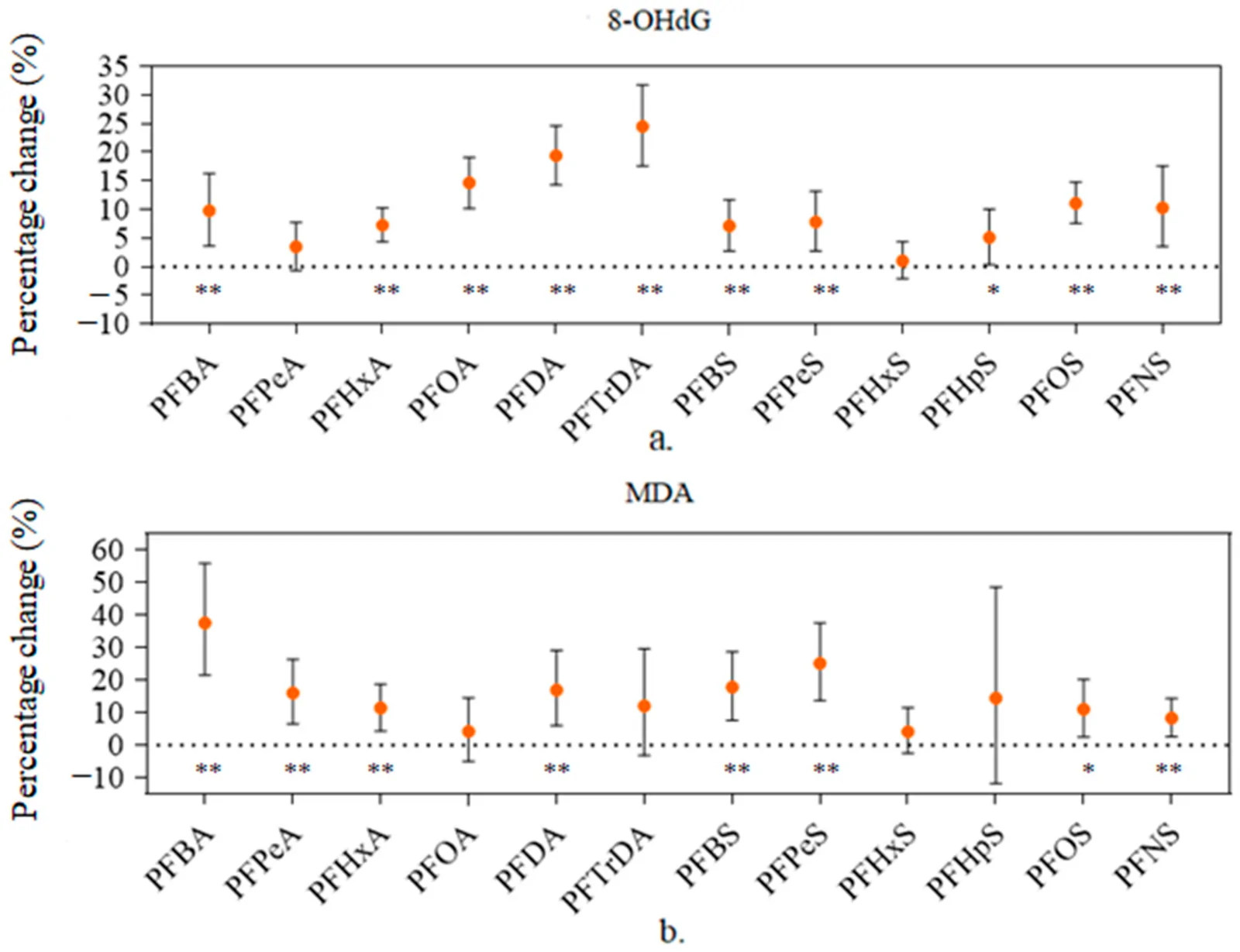
Estimated percent changes (95% CI) in urinary creatinine-adjusted 8-OHdG (**a**) and MDA. (**b**) Per unit increase in ln-transformed urinary creatinine-adjusted PFASs among all workers in the waste recovery plant utilizing LME models, incorporating modifications for gender, age, tobacco use, BMI, employment role, arterial pressure, glucose levels, and lipid profiles. Abbreviations: confidence interval, CI; 8-hydroxydeoxyguanosine, 8-OHdG; malondialdehyde, MDA; ** *p* < 0.01; * *p* < 0.05.

In this occupational cohort, the mean urinary levels of 8-OHdG and MDA were 25.4 ± 9.83 μg/L and 76.2 ± 67.9 μg/L. 8-OHdG and MDA mainly exhibit significant exposure–response relationships with multiple urinary short-chain PFASs (Supplementary Materials, Table S9). For DNA damage, a one-unit increase in ln-transformed urinary concentrations of PFBA, PFHxA, PFOA, PFDA, PFBS, PFPeS, PFHpS, and PFOS was associated with a 5.13% (95% CI: 0.30–10.1%) to 24.5% (95% CI: 17.6–31.8%) increase in urinary 8-OHdG levels (*p* < 0.05) (Figure 4a). For lipid peroxidation, a one-unit increase in ln-transformed urinary concentrations of PFBA, PFPeA, PFHxA, PFBS, PFPeS and PFOS corresponded to an 8.33% (95% CI: 2.66–14.3%) to 37.6% (95% CI: 21.5–55.9%) increase in urinary MDA levels (*p* < 0.05) (Figure 4b).

### 3.5. Associations Between Size-Resolved PM-Bound PFASs and Urinary Oxidative Stress Biomarkers

Further LME model analysis shows that several short-chain PFASs, including PFBA, PFPeA, PFHxA, PFBS and PFHxS, were significantly associated with DNA oxidative damage, with the magnitude of these associations varying across particle size fractions (Supplementary Materials, Table S10) (Figure 3b). In contrast, except for PFOS, long-chain PFASs showed no significant association with 8-OHdG (*p* > 0.05). Overall, urinary 8-OHdG concentrations were predominantly associated with ∑PFCAs in the PM_4.7–5.8_ fraction and ∑PFSAs in the PM_9.0–10_. A one-unit increase in the ln-transformed ADI of these combined PFASs was associated with a 10.2–12.6% increase in urinary 8-OHdG (*p* < 0.01). Urinary MDA levels were predominantly associated with PFHxA and several long-chain PFCAs in the PM_5.8–9.0_ (Supplementary Materials, Table S11). A one-unit increase in the ln-transformed ADI of these PFASs corresponded to an 8.0–14.6% increase in urinary MDA levels (*p* < 0.05) (Figure 3c).

## 4. Discussion

In this 45-day panel study, we comprehensively characterized external and internal exposure to size-resolved PM-bound PFASs and their links to DNA and lipid oxidative damage biomarkers. Our findings expand current understanding of PFASs exposure and health effect and PM toxicity.

Previous studies have suggested that long-chain PFASs preferentially partition into PM due to their higher octanol–air partition coefficients [51]. However, in the present in occupational environments, PFASs in PM were predominantly composed of short-chain PFASs, consistent with their increased industrial use as substitutes for long-chain compounds and their potential formation via precursor degradation pathways [52]. Notably, the majority of PFAS mass was associated with the PM_2.1–10_ fraction, in contrast to outdoor environments where PFASs are more commonly associated with finer particles [15,53]. This pattern likely reflects source-specific characteristics and the resuspension of contaminated dust within the setting. The PM_2.1–10_ fraction was the primary contributor to respiratory deposition and exhibited stronger associations with urinary PFASs concentrations than the PM_2.1_, highlighting the critical role of coarse particles in exposure assessments within occupational environments.

Short-chain PFASs also dominated urinary PFASs profiles, which may be attributed to their higher environmental exposure levels [54,55], faster renal clearance compared with long-chain PFASs [56] and potential formation through metabolic transformation of fluorinated precursors. The elevated concentrations of short-chain PFCAs in both PM and urine when compared to those observed under non-occupational environments underscore the increased exposure burden in this occupational setting [15,57,58]. Urinary PFASs concentrations vary according to occupation, sex, age, and lifestyle habit, reflecting the combined influence of exposure sources and physiological characteristics. Sex-specific differences may be explained by physiological elimination pathways in women, such as menstruation and lactation, resulting in generally higher PFASs concentrations in males than in females [59]. The fair-to-excellent ICCs observed for urinary short-chain PFASs over the 45-day period suggest that a single spot urine sample can reliably reflect recent exposure for these compounds in populations with relatively stable lifestyles and work patterns, thereby reducing exposure misclassification in large-scale studies [47]. The poor ICCs observed for long-chain congeners further indicate that urine is a more suitable matrix for assessing recent exposure to short-chain PFASs rather than long-chain compounds [7].

8-OHdG and MDA are well-established OSBs commonly used to reflect health status and reveal associations with disease risk [60,61]. The urinary 8-OHdG concentration observed in our study (4.77 μg/g-Cre; 25.4 μg/L) was similar to that in the urine of Chinese e-waste recycling [62], but was 1.3–4 times greater than that measured in residents of South China [63] and Iran [63,64], underscoring the elevated health risks associated with occupational exposure. Previous research has linked serum concentrations of long-chain PFASs to oxidative stress [36,37,38,65]. A key finding of this study is the significant association between urinary short-chain PFASs and elevated OSBs, providing preliminary evidence that short-chain PFASs may also pose health risks in occupational settings. However, these findings should be considered exploratory, and further comparative toxicological assessments are needed to establish their biological significance. Because urine is not an optimal biomonitoring matrix for assessing exposure to long-chain PFASs, the observed associations between the urinary concentrations of certain long-chain PFASs (e.g., PFOA and PFOS) and OSBs should be interpreted with caution and confirmed using paired serum measurements.

Most importantly, we observed suggestive quantitative associations between inhaled size-resolved PFASs and urinary OSBs, which warrant further confirmation. The finding that PFASs in specific size fractions (PM_4.7–5.8_ and PM_9.0–10_ for 8-OHdG; PM_5.8–9.0_ for MDA) are associated with different oxidative damage endpoints emphasize once again the importance of coarse particles in occupational exposure assessments and suggests that particle size influencing deposition site, deposition dose and subsequent bioavailability may play a role in these coarse-related health effects. These associations remained significant despite the relatively minor contribution of inhalation to total daily PFASs intake and the low noncarcinogenic HQ. These findings suggest that PM-bound PFASs may be associated with molecular-level damage even at relatively low exposure concentrations, potentially through interactions with pulmonary cells that trigger both local and systemic oxidative stress [66,67]. Such subclinical damage may represent an early oxidative-stress-related response that has been linked to a range of adverse health outcomes, including metabolic and cardiovascular disorders [67,68]. Previous studies have underscored the importance of monitoring and controlling hazardous constituents in PM, rather than focusing solely on total particulate mass [69]. In addition to conventional PM components, such as heavy metals and PAHs [18,69,70], we think that greater attention should be directed toward trace persistent organic pollutants in specific working environments, as these may also represent important contributors to PM-related health effects.

This study has several strengths, including a repeated-measures panel design, comprehensive size-resolved exposure assessment, and longitudinal urinary sampling. However, its several limitations should be acknowledged. First, PFASs isomers were not analyzed, despite their potentially distinct toxicokinetic properties. Second, dietary intake, dust ingestion, gaseous PFASs were not quantified, which may have introduced uncertainty into exposure estimates. Third, the modest sample size limited the statistical power for detailed subgroup analyses. Although repeated measurements (280 urine samples) increased the statistical power of the longitudinal analyses, between-individual variability may have been underestimated. Therefore, caution is warranted when extrapolating these findings to other populations, and future studies involving larger independent cohorts are needed. Furthermore, urinary 8-OHdG and MDA are nonspecific biomarkers of oxidative stress. Their levels can be influenced by multiple factors, including smoking, diet, obesity, inflammation, physical workload, and metabolic status. Although we adjusted for several covariates (e.g., smoking status, BMI, blood pressure, glucose levels, lipid profiles) in the LME models, residual confounding cannot be completely excluded. More importantly, the airborne PM mixture was not chemically characterized beyond PFASs. Therefore, other constituents, such as heavy metals and polycyclic aromatic hydrocarbons, which are known to induce oxidative stress, may also have contributed to the observed associations. Therefore, our findings should be interpreted as statistical associations observed within a complex occupational exposure environment rather than as evidence that PFASs independently cause oxidative stress. Establishing a causal relationship will require future studies incorporating more comprehensive exposure characterization, larger populations, and mechanistically informed experimental designs.

## 5. Conclusions

Occupational exposure was associated with elevated internal and external levels of PFASs, as well as increased OSBs. Short-chain PFASs predominate in both PM and urine. In this cohort with stable lifestyles and work patterns, a first morning void urine sampling proved to be a reliable indicator of short-chain PFASs exposure over the 45-day monitoring period. Notably, the PM_2.1–10_ fraction exhibited stronger associations with urinary PFAS concentrations than the PM_2.1_. Importantly, we observed potential exposure–response relationships between inhaled size-resolved PM-bound PFASs and urinary short-chain PFASs with OSBs. Inhalation exposure to PFASs in the PM_4.7–5.8_ and PM_9.0–10_ showed stronger associations with DNA oxidative damage, whereas exposure to PFASs in the PM_5.8–9.0_ was more strongly associated with lipid peroxidation. These findings suggest potential associations between PM_2.1–10_-bound short-chain PFASs and elevated OSBs. However, because of the exploratory nature of our analyses and the lack of multiple-comparison correction, these results should be interpreted cautiously and are not conclusive evidence of causality. Future research is needed to validate our observations and elucidate underlying mechanisms.

## Figures and Tables

**Table 1:** Demographic characteristics of the study population.

Job Assignment	Gender	Age (Year)	Working Place	Exposure Time (h)	Number (%)
Driver	Male	40–50	Uncertain	1	4 (20)
Sorter	Female	40–60	Warehouse	10	7 (35)
Stevedore	Male	48, 53	Warehouse	10	2 (10)
Manager	Female	24–27	Office	0	2 (10)
Logistics	Female & Male	51–58	Logistics Room	0	2 (10)
Other	Male	25–60	Warehouse	10	3 (15)
Job tenure (year)					Number (%)
0–1					6 (30)
2–4					14 (70)
Height (cm)	Number (%)	Weight (kg)	Number (%)	Body weight index (Kg/m^2^)	Number (%)
140–160	12 (60)	40–60	11 (55)	Overweight (24–28)	13 (65)
160–180	8 (40)	60–110	9 (45)	Normal (<24)	7 (35)
Blood pressure	Number (%)	Blood fat	Number (%)	Blood sugar	Number (%)
High	4 (20)	High	7 (35)	High	2 (10)
Normal	16 (80)	Normal	13 (65)	Normal	18 (90)

**Table 2:** Distribution of perfluorinated and polyfluoroalkyl substances concentrations (mean/median, ng/L) across various job roles and age demographics in the waste recycling plant.

	Job Assignment					Job Tenure (Year)			
	Driver	Sorter	Stevedore	Manager	*p*	<1	1–2	n = 4	*p*
PFBA	78.8/71.3	75.9/74.8	55.4/56.7	44.9/45.7	**	77.3/76.9	65.2/60.4	70.9/64.6	
PFPeA	1023/830	673/485	1050/924	645/366	**	736/342	692/576	944/957	*
PFHxA	18.3/7.94	6.70/4.59	4.45/4.49	4.17/3.53	**	6.59/4.94	17.1/6.39	19.4/5.55	**
PFOA	90.7/77.1	71.0/55.9	69.6/65.6	54.9/49.3	**	59.4/42.0	64.2/59.2	77.7/63.8	
PFDA	2.66/1.93	1.91/1.28	2.63/1.52	1.75/1.22		2.31/1.43	1.70/1.28	2.18/1.79	
PFTrDA	2.14/2.40	2.00/2.15	2.30/2.44	2.05/2.36		2.04/2.25	2.15/2.31	2.88/2.44	
PFBS	18.5/13.7	20.6/16.1	52.4/25.8	12.7/12.4	**	16.2/13.2	15.4/12.0	31.9/19.7	**
PFPeS	5.54/3.69	4.14/1.65	4.06/2.34	5.98/2.61		4.24/1.34	7.56/3.94	7.02/2.89	**
PFHxS	268/160	127/53.4	141/122	130/39.0	**	203/151	169/69.9	217/71.4	
PFHpS	10.4/6.15	4.83/2.76	5.16/4.32	4.26/2.73	**	5.53/4.85	6.96/2.56	6.42/2.75	
PFOS	12.9/10.5	10.2/8.90	11.8/11.9	13.4/11.5		12.2/12.0	9.46/7.02	12.2/9.95	
PFNS	1.03/0.43	1.02/0.30	1.33/0.30	1.56/0.78		1.29/0.42	1.06/0.39	1.09/0.46	
ΣPFCAs	1215/980	831/609	1184/1135	753/481	**	884/498	843/727	1116/1012	
ΣPFSAs	321/183	168/97.0	216/167	168/77.2	**	242/176	209/107	276/130	

Variations among two or more groups were assessed through general linear models. **: *p* < 0.01. *: *p* < 0.05.

**Table 3:** Components of variance and intraclass correlation coefficients for the natural logarithm of urinary creatinine-adjusted levels of various PFASs among employees with diverse job roles over a span of 45 continuous days.

		PFBA	PFPeA	PFHxA	PFOA	PFDA	PFTrDA	PFBS	PFPeS	PFHxS	PFHpS	PFOS	PFNS
Driver	W-σ^2^	0.25/0.14	0.38/0.78	0.98/1.24	1.18/0.75	2.48/1.43	1.9C3/2.563	4.91/4.06	5.71/7.79	3.38/0.21	3.90/4.96	7.13/6.16	4.68/5.14
	B-σ^2^	1.01/0.91	2.41/3.81	8.64/3.43	2.12/2.09	0.95/0.95	0.94/0.13	0.50/0.02	3.85/1.45	4.24/1.06	0.42/0.63	6.16/0.82	0.48/0.70
	ICCs	0.80/0.87	0.86/0.83	0.90/0.73	0.64/0.74	0.28/0.40	0.33/0.05	0.09/0.01	0.40/0.16	0.56/0.54	0.10/0.11	0.46/0.29	0.09/0.12
Sorter	W-σ^2^	0.35/0.71	1.25/2.43	3.89/3.39	0.81/0.85	3.01/2.34	5.36/4.80	0.79/0.87	4.09/4.56	1.87/0.19	2.02/1.73	2.31/0.09	3.39/4.19
	B-σ^2^	0.54/0.98	28.7/24.3	2.69/3.14	7.92/6.44	0.31/0.44	0.594/0.11	0.85/0.00	3.02/2.24	9.50/1.06	1.31/0.93	4.27/0.87	1.18/0.89
	ICCs	0.61/0.58	0.96/0.91	0.41/0.48	0.91/0.88	0.09/0.16	0.10/0.02	0.52/0.01	0.42/0.33	0.84/0.78	0.39/0.35/	0.65/0.69	0.25/0.17
Stevedore	W-σ^2^	0.10/0.31	0.21/0.52	1.14/1.95	0.37/0.28	2.21/1.90	1.31/1.31	0.25/0.73	4.25/4.96	0.53/0.32	1.43/1.36	3.07/0.34	2.27/2.71
	B-σ^2^	0.23/0.01	0.78/1.60	2.84/3.27	3.54/2.87	0.38/1.07	0.005/0.08	0.04/0.00	4.98/4.17	3.36/0.53	1.40/0.93	0.13/0.68	0.37/0.13
	ICCs	0.69/0.03	0.79/0.76	0.71/0.63	0.91/0.91	0.15/0.36	0.004/0.05	0.13/0.00	0.54/0.46	0.86/0.78	0.49/0.41	0.04/0.00	0.14/0.04
Manager	W-σ^2^	0.82/0.45	0.43/0.98	2.34/1.26	0.60/0.32	0.51/0.45	1.26/1.36	1.56/0.76	2.03/2.82	1.92/0.59	1.37/1.18	5.43/0.91	2.57/2.78
	B-σ^2^	0.44/3.48	0.04/2.00	9.38/18.2	2.89/0.25	1.97/0.04	1.70/0.01	3.79/0.11	2.03/2.82	11.4/0.09	0.24/0.51	2.23/1.76	1.75/0.01
	ICCs	0.35/0.88	0.09/0.67	0.80/0.94	0.83/0.43	0.79/0.08	0.60/0.01	0.71/0.13	0.70/0.80	0.86/0.94	0.15/0.30	0.29/0.02	0.40/0.01

Abbreviations: intraclass correlation coefficient, ICCs; within-individual variance, W-σ^2^; between-individual variance, B-σ^2^; adjusted for urinary creatinine, Cre-adjusted; without creatinine adjustment, Un-adjusted.

## Data Availability

The original contributions presented in this study are included in the article/Supplementary Material. Further inquiries can be directed to the corresponding author.

## Supplementary Materials

The following supporting information can be downloaded at https://www.mdpi.com/article/10.3390/toxics14080682/s1, Table S1: Instrument parameters used to measure PFASs using LC-MSMS; Table S2: The calculation method of basal metabolic rate (BMR) for people with different ages. Table S3: The long-term respiratory coefficient (A) for people of different ages. Table S4: Quality assurance/quality control (QA/QC) results for PFASs in air PM_10_; Table S5: Quality assurance/quality control (QA/QC) results for PFASs in urine; Table S6: Average mass concentrations (pg/m^3^) of PFASs in air PM_10_ in the waste recycle plant; Table S7: Concentrations (ng/L) of PFASs in urine samples of waste recycling workers; Table S8: Regression coefficient (β) for ln-transformed urinary creatinine-adjusted PFASs (pg/g-Cre) with ln-transformed daily intake of size-resolved PM_10_-bound PFASs (pg/day) via inhalation among warehouse workers by linear mixed-effect models (LME); Table S9: Regression coefficient (β) for the ln-transformed urinary creatinine-adjusted 8-OHdG and MDA (pg/g-Cre) with the ln-transformed urinary creatinine-adjusted PFASs (pg/g-Cre) among warehouse workers by linear mixed-effect models (LME); Table S10: Regression coefficient (β) for ln-transformed urinary creatinine-adjusted 8-OHdG (pg/g-Cre) with ln-transformed daily intake (pg/day) of size-resolved PM_10_-bound PFASs via inhalation among warehouse workers by linear mixed-effect models (LME);Table S11: Regression coefficient (β) for ln-transformed urinary creatinine-adjusted MDA (pg/g-Cre) with ln-transformed daily intake (pg/day) of size-resolved PM_10_-bound PFASs via inhalation among warehouse workers by linear mixed-effect models (LME); Figure S1: The relative contributions (%) of PM with different sizes to total PFASs deposition in the HA, TB and PA of respiratory tract. Abbreviations: head airway, HA; tracheobronchial, TB; and pulmonary, PA. Un-deposition is the fraction of PFASs not deposited in respiratory tract during respiration. The % is for mass concentrations; Figure S2: Concentration (μg/L and μg/g-Cre) of PFASs in urine of different gender (a,b) and age groups (c,d) in the waste recycling plant; Figure S3: A polar heatmap (%) illustrating the likelihood of intraclass correlation coefficients exceeding 0.40 for ln-transformed concentrations of PFASs adjusted for urinary creatinine, analyzed by sorters across randomly chosen consecutive sampling days through two-way random-effects models. The references [45,71] cite in Supplementary Materials.
