# Supplementary material for: Size-Resolved PM10-Bound PFASs: Respiratory Deposition and Association with Oxidative Stress Biomarkers Among Industrial Waste Recycling Workers
Source: Toxics. 2026 Aug 3;14(8):682. doi: 10.3390/toxics14080682 (PMC13517902; doi:10.3390/toxics14080682)
Supplement: Supplementary file 1 [file toxics-14-00682-s001.zip › toxics-4309192-supplementary.pdf]

SUPPORTING INFORMATION

Chemicals and reagents.

The 26 target PFASs were detected, including perfluorobutanoic acid (PFBA), perfluoropentanoic acid (PFPeA), perfluorohexanoic acid (PFHxA), perfluoroheptanoic acid (PFHpA), perfluorooctanoic acid (PFOA), perfluorononanoic acid (PFNA), perfluorodecanoic acid (PFDA), Perfluoroundecanoic acid (PFUnDA), perfluorododecanoic acid (PFDoA), perfluorotridecanoic acid (PFTrDA), perfluorotetradecanoic acid (PFTeDA), perfluorobutanesulfonic acid (PFBS), perfluoropentanesulfonic acid (PFPS), perfluorohexanesulfonic acid (PFHxS), perfluoroheptanesulfonic acid (PFHpS), PFOS, perfluorononanesulfonic acid (PFNS), perfluorodecanesulfonic acid (PFDS), n-methylperfluorooctanesulfonamide (N-MeFOSA) (N-MeFOSA), n-ethylperfluorooctanesulfonamide (N-EtFOSA), n-methylperfluorooctanesulfonamidoacetic acid (N-MeFOSAA), perfluorooctanesulfonamidoacetic acid (N-EtFOSAA), 1H,1H,2H,2H-perfluorohexane sulfonate acid (4:2FTSA), 1H,1H,2H,2H-Perfluorooctane sulfonate acid (6:2 FTSA), 1H,1H,2H,2H-perfluorodecane sulfonate acid (8:2 FTSA) and 1H,1H,2H,2H-Perfluorododecane sulfonate acid (10:2 FTSA) . $^{13}C_3$-PFBA, $^{13}C_5$-PFPeA,$^{13}C_8$-PFOA, $^{13}C_9$-PFNA, $^{13}C_2$-PFDA, $^{13}C_2$-PFUdA, $^{13}C_2$-PFHxDA, $^{13}C_3$-PFHxS, $^{13}C_8$-PFOS, and $^{13}C_8$-PFOS were used as internal standards. All the native and their isotope-labeled internal standards were purchased from Cambridge Isotope Laboratories (MA, USA) and Wellington Laboratories (Guelph, ON, Canada).

Standards of 8-OHdG and creatinine, Malondialdehyde tetrabutylammonium salt and 2,4-dinitrophenylhydrazine and formic acid (98.2 %) were obtained from Sigma–Aldrich (St. Louis, MO, USA). Internal standards of $^{15}N_5$-8-OHdG and d_5-Creatinine and 1,3-[$^2H_2$]-1,1,3,3-tetraethoxypropane (d_2-TEP) were purchased from Cambridge Isotope Laboratories (Andover, MA, USA). [$^2H_2$] Malondialdehyde (d_2-MDA) was obtained by hydrolyzing d_2-TEP in 0.02 N HCl for 2 h at 25-28°C according to a previous study [71]. HPLC-grade methyl tert-butyl ether (MTBE), ammonium acetate, methanol, acetonitrile and water were obtained from Fisher Scientific (PA,USA). Tetra-n-butylammonium hydrogen sulfate

(TBAH,≥99 %) were purchased from Nanjing Duly Biotechnology Co., Ltd. Sodium carbonate was obtained from Aladdin (≥ 99.999%). Sodium bicarbonate was purchased from Macklin (≥ 99.0%).

Sample Preparation.

PM_{10}-bound PFASs In brief, a quarter filter sample was spiked with 1 ng of mixed solution of internal standards (20 μL, 50 ng/mL) in a 15 mL PP tube, then 4 mL of MTBE was added. The solution was mixed well and extracted by ultrasonic for 15 minutes. After that, the mixture was shaken for 30 min at 200 rpm/min followed by centrifugation at 8000 rpm for 10 min. The supernatant organic phase was transferred to a new 15 mL PP tube, and the residue was extracted twice with 4 mL of MTBE, as described above. All of the supernatants were combined and evaporated to near dryness under a gentle flow of nitrogen gas at room temperature, and 300 μL of methanol was then added. Finally, the eluent was filtered through a 0.22 μm nylon filter into a plastic injection vial for instrumental analysis.

Urinary PFASs In brief, 2 mL of urine sample was spiked with 1 ng of mixed solution of internal standards (20 μL, 50 ng/mL) in a 15 mL PP tube, the internal standard and sample was shaken and mixed, then 2 mL sodium carbonate/sodium bicarbonate buffer solution (0.25 M,pH=10), 1 mL TBAH (0.5M) and 4 mL of MTBE were added. The solution was mixed well and extracted by ultrasonic for 15 minutes. After that, the mixture was shaken for 30 min at 200 rpm/min followed by centrifugation at 8000 rpm for 10 min. The supernatant organic phase was transferred to a new 15 mL PP tube, and the residue was extracted twice with 4 mL of MTBE, as described above. All of the supernatants were combined and evaporated to near dryness under a gentle flow of nitrogen gas at room temperature, and 300 μL of methanol was then added. Finally,

the eluent was filtered through a 0.22 μm nylon filter into a plastic injection vial for instrumental analysis.

Urinary creatinine, 8-OHdG and MDA Sample Preparation of urinary creatinine, 8-OHdG and MDA can be found in our previous study.

Instrument Analysis.

Quantitative analysis of the 26 target PFASs were determined using an ultraperformance liquid chromatography system (Nexera-XZ UPLC, Shimadzu Corporation Inc.Kyoto, JP) coupled with triple quadrupole mass spectrometer (AB SCIEX TRIPLE QUAD 6500, Foster City, CA, USA) in a negative electrospray ionization mode. The mobile phase consisted of 2 mM ammonium acetate (A) and methanol (B). A total flow rate of 250 μL/min was used. The separation of the PFASs was achieved by a Betasil C18 column (Thermo Scientific, USA; 100 × 2.1 mm, 5 μm) and the column oven temperature was set at 40 ºC. The gradient was set as follows: initial condition, 80% of A; held for 1.0 min with 80% of A; 1.0− 6.0 min, decreased linearly to 5% of A; 6.0− 8.0 min, held with 5% of A; 8.0− 8.1 min, rapidly returned to 80% of A; 8.1− 10.0 min, held with 80% of A. The injection volume was 2 μL. More instrumental parameters were set as follows: ion spray voltage, 4500 V; ion source temperature, 350 ºC. The HPLC-MS/MS operation parameters are presented in Table S1.

The HPLC-MS/MS operation parameters for urinary creatinine, 8-OHdG and MDA can be found in our previous study.

Table S1. Instrument parameters used to measure PFASs using LC-MSMS.

substance	Q1 （m/z）	Q3 （m/z）	CV （V）	EP （V）	CE （V）	CXP （V）	CUP （mL/min）	CAD （mL/min）	GAS1 （mL/min）	GAS2 （mL/min）
PFBA	213	169	-15	-10	-13	-15	20	9	30	40
PFPeA	263	219	-20	-10	-12	-15	20	9	30	40
PFHxA	313	269	-20	-10	-13	-13	20	9	30	40
PFHpA	363	319	-15	-10	-14	-20	20	9	30	40
PFOA	413	369	-20	-11	-15	-18	20	9	30	40
PFNA	463	419	-15	-10	-16	-20	20	9	30	40
PFDA	513	469	-20	-11	-16	-10	20	9	30	40
PFUdA	563	519	-15	-12	-19	-10	20	9	30	40
PFDoA	613	569	-15	-13	-19	-10	20	9	30	40
PFTrDA	663	619	-15	-10	-20	-10	20	9	30	40
PFTeDA	713	669	-15	-10	-20	-10	20	9	30	40
PFBS	299	99	-15	-10	-36	-10	20	9	30	40
PFPeS	349	99	-15	-10	-38	-10	20	9	30	40
PFHxS	399	99	-15	-7	-75	-10	20	9	30	40
PFHpS	449	99	-15	-10	-80	-10	20	9	30	40
PFOS	499	99	-15	-7	-100	-10	20	9	30	40
PFNS	549	99	-15	-8	-110	-10	20	9	30	40
PFDS	599	99	-15	-10	-110	-10	20	9	30	40
N-MeFOSA	512	169	-20	-10	-40	-10	20	9	30	40
N-EtFOSA	526	169	-20	-10	-36	-10	20	9	30	40
N-MeFOSAA	570	419	-20	-10	-30	-10	20	9	30	40
N-EtFOSAA	584	419	-20	-10	-28	-10	20	9	30	40
4：2 FTSA	327	307	-20	-10	-28	-10	20	9	30	40
6：2 FTSA	427	407	-20	-10	-34	-15	20	9	30	40
8：2 FTSA	527	507	-20	-10	-38	-15	20	9	30	40
10：2 FTSA	627	607	-20	-10	-42	-15	20	9	30	40
$^{13}C_3$-PFBA	216	172	-10	-10	-13	-15	20	9	30	40
$^{13}C_5$-PFPeA	268	223	-10	-10	-12	-10	20	9	30	40
$^{13}C_2$-PFHxA	315	270	-10	-10	-13	-10	20	9	30	40
$^{13}C_7$-PFHpA	370	325	-10	-10	-14	-10	20	9	30	40
$^{13}C_8$-PFOA	421	376	-10	-10	-15	-10	20	9	30	40
$^{13}C_9$-PFNA	472	427	-10	-10	-15	-10	20	9	30	40
$^{13}C_2$-PFDA	515	470	-10	-10	-15	-10	20	9	30	40
$^{13}C_2$-PFUdA	565	520	-10	-10	-17	-10	20	9	30	40
$^{13}C_2$-PFDoA	615	570	-15	-10	-19	-10	20	9	30	40
$^{13}C_2$-PFHxDA	815	770	-15	-10	-24	-15	20	9	30	40
$^{13}C_2$-PFTeDA	715	670	-10	-10	-20	-10	20	9	30	40
$^{13}C_3$-PFHxS	402	99	-15	-10	-75	-15	20	9	30	40
$^{13}C_8$-PFOS	507	99	-15	-10	-100	-15	20	9	30	40
D_3-N-MeFOSA	515	169	-20	-10	-32	-15	20	9	30	40

D_5-N-EtFOSA	531	169	-20	-10	-37	-15	20	9	30	40
D_3-N-MeFOSAA	573	419	-20	-10	-28	-20	20	9	30	40
D_5-N-EtFOSAA	589	419	-20	-10	-29	-20	20	9	30	40
D_4-4:2 FTSA	329	309	-20	-10	-28	-15	20	9	30	40
D_4-6:2 FTSA	433	412	-20	-10	-33	-15	20	9	30	40
D_4-8:2 FTSA	533	512	-20	-10	-40	-20	20	9	30	40
D_4-10:2 FTSA	633	612	-20	-10	-45	-20	20	9	30	40

Abbreviations: Parent ion, Q1; Product ion, Q3; Cone voltage, CV; Entrance voltage, EP; Collision energy, CE; Collision export voltage, CXP; Curtain gas, CUP; Collision gas, CAD; Ion source gas 1, GAS1; Ion source gas 2, GAS2.

Model and data analysis The inhalation rate (R_{inh}: m^3/h) among workers were estimated based on basal metabolic rate (BMR) (kJ/d) [45] as follows:

$$\text{Rinh} = \frac{BRM \times E \times VQ \times A}{1000},$$

where E is oxygen consumption per unit energy metabolism, and usually is 0.05 L/kJ; VQ is ventilation equivalent, and usually is 27; and A is long term respiratory coefficient associated with age and sex[45]; more detailed information about BMR and A can be seen in Table S2 and Table S3.

Table S2. The calculation method of basal metabolic rate (BMR) for people with different ages.

Age	Male	Female
18 ≤Age＜30	BMR=63BW＋2896	BMR=62BW＋2036
30 ≤Age＜60	BMR=48BW＋3653	BMR=34BW＋3528
60 ≤Age	BMR=49BW＋2459	BMR=38BW＋2755

Note: BW is body weight, unit is kg, data were collected from questionnaire.

Table S3. The long-term respiratory coefficient (A) for people of different ages.

Age	Male	Female
18≤Age＜23	1.9	1.6
23≤Age＜35	1.8	1.6
35≤Age＜51	1.8	1.5
51≤Age＜65	1.7	1.5
65≤Age＜75	1.8	1.5
75≤Age	1.9	1.6

Table S4. Quality assurance/quality control (QA/QC) results for PFASs in air PM_{10}.

Compound	Internal standard	Carbon atoms	LOD （ng/L）	Sample recovery （IS） (%)	Blank spike recovery (IS) (%)	Matrix spike recovery (IS)(%)	Blank spike recovery (TS) (%)	Matrix spike recovery(TS)(%)
PFBA	$^{13}C_3$-PFBA	4	7	80 ± 12	82 ± 10	79 ± 8	78 ±3	79 ± 3
PFPeA	$^{13}C_5$-PFPeA	5	2	79 ± 13	82 ± 10	78 ± 13	77 ± 2	78 ± 2
PFHxA	$^{13}C_2$-PFHxA	6	2	73 ± 13	78 ± 8	73 ± 14	83 ± 6	84 ± 6
PFHpA	$^{13}C_7$-PFHpA	7	1	73 ± 15	78 ± 9	74 ± 13	77 ±4	78 ± 4
PFOA	$^{13}C_8$-PFOA	8	2	73 ± 15	78 ± 9	74 ± 12	77 ± 4	79 ± 4
PFNA	$^{13}C_9$-PFNA	9	1	74 ± 14	85 ± 8	81 ± 14	75 ± 4	76 ± 4
PFDA	$^{13}C_2$-PFDA	10	2	71 ± 15	88 ± 10	83 ± 14	70 ± 6	72 ± 6
PFBS	$^{13}C_3$-PFHxS	4	1	71 ± 16	74 ± 8	74 ± 8	77 ± 5	78 ± 5
PFHxS	$^{13}C_3$-PFHxS	6	1	71 ± 16	74 ± 8	72 ± 8	72 ± 4	76 ± 4
PFOS	$^{13}C_8$-PFOS	8	1	76 ± 15	90 ± 8	72 ± 8	75 ± 3	77 ± 3

Abbreviations: internal standard, IS; target standard, TS. Mean ± Standard deviation

Table S5. Quality assurance/quality control (QA/QC) results for PFASs in urine.

Compound	Internal standard	Carbon atoms	LOD （ng/mL）	Sample recovery （IS）(%)	Blank spike recovery (IS) (%)	Matrix spike recovery (IS) (%)	Blank spike recovery (TS) (%)	Matrix spike recovery(TS)(%)
PFBA	$^{13}C_3$-PFBA	4	0.007	61 ± 15	84 ± 4	69 ± 4	73 ± 4	70 ± 4
PFPeA	$^{13}C_5$-PFPeA	5	0.002	97 ± 17	96 ± 7	103 ± 7	71 ± 5	62 ± 6
PFHxA	$^{13}C_2$-PFHxA	6	0.002	92 ± 17	96 ± 8	92 ± 11	73 ± 5	75 ± 5
PFOA	$^{13}C_8$-PFOA	8	0.002	93 ± 17	94 ± 6	92 ± 4	69 ± 4	76 ± 3
PFDA	$^{13}C_2$-PFDA	10	0.002	82 ± 26	97 ± 8	92 ± 5	73 ± 6	73 ± 4
PFTrDA	$^{13}C_2$-PFHxDA	13	0.001	82 ± 18	102 ± 10	90 ± 5	72 ± 6	59 ± 3
PFBS	$^{13}C_3$-PFHxS	4	0.001	88 ± 15	87 ± 11	93 ± 4	68 ± 8	88 ± 2
PFPeS	$^{13}C_3$-PFHxS	5	0.001	88 ± 15	87 ± 11	93 ± 4	69 ± 7	71 ± 6
PFHxS	$^{13}C_3$-PFHxS	6	0.001	88 ± 15	87 ± 11	93 ± 4	72 ± 7	87 ± 26
PFHpS	$^{13}C_8$-PFOS	7	0.002	89 ± 17	91 ± 9	91 ± 3	70 ± 6	79 ± 6
PFOS	$^{13}C_8$-PFOS	8	0.001	89 ± 17	91 ± 9	91 ± 3	68 ± 4	79 ± 4
PFNS	$^{13}C_8$-PFOS	9	0.001	89 ± 17	91 ± 9	91 ± 3	72 ± 5	72 ± 3

Abbreviations: internal standard, IS; target standard, TS. Mean ± Standard deviation

Table S6. Average mass concentrations (pg/m^3) of PFASs in air PM_{10} in the waste recycle plant.

Species	Detection frequency（%）	PM_{10}							
		$PM_{0.43–0.65}$	$PM_{0.65–1.1}$	$PM_{1.1–2.1}$	$PM_{2.1–3.3}$	$PM_{3.3–4.7}$	$PM_{4.7–5.8}$	$PM_{5.8–9.0}$	$PM_{9.0-10}$
PFBA	94.0	2.45	3.18	4.04	4.73	4.82	5.90	5.27	5.31
PFPeA	82.9	1.23	1.80	1.07	1.04	0.86	1.01	2.94	0.77
PFHxA	95.7	1.89	1.13	1.10	1.29	0.99	1.07	1.31	0.98
PFHpA	63.2	0.39	2.34	0.71	1.01	0.71	1.05	1.77	0.87
PFOA	92.3	1.44	1.34	1.07	0.98	0.90	0.99	2.07	1.31
PFNA	52.1	0.12	0.32	0.18	0.24	0.35	0.12	0.50	0.09
PFDA	69.2	0.32	0.29	0.41	0.48	0.45	0.30	1.04	0.47
ΣPFCAs		7.86	10.4	8.57	9.75	9.09	10.4	14.9	9.78
PFBS	63.2	0.69	0.59	0.56	1.16	1.01	0.92	2.03	3.65
PFHxS	63.2	0.39	0.41	0.38	0.36	0.65	0.39	0.51	0.47
PFOS	70.9	0.65	0.62	1.70	1.52	1.22	0.45	1.64	0.48
ΣPFSAs		1.73	1.61	2.63	3.03	2.87	1.76	4.17	4.59

Table S7. Concentrations (ng/L) of PFASs in urine samples of waste recycling workers.

Species	Detection frequency（%）	Concentrations （ng/L）						
		Mean	Standard deviation	Min	P25	P50	P75	Max
PFBA	99	70.5	41.2	NF	46.9	63.6	88.9	321
PFPeA	99	825	705	NF	306	690	1145	3840
PFHxA	95	16.0	90.2	NF	3.03	5.67	10.3	1462
PFOA	99	69.9	51.0	NF	33.5	59.3	88.3	338
PFDA	66	2.06	3.15	NF	NF	1.55	2.84	25.8
PFTrDA	53	2.49	6.72	NF	NF	NF	3.96	107
ΣPFCAs		986	742	NF	423	840	1297	4278
PFBS	94	23.6	45.9	NF	9.20	14.6	38.1	588
PFPeS	66	6.62	15.0	NF	NF	2.62	6.81	188
PFHxS	99	200	354	NF	29.1	77.4	488	3547
PFHpS	59	6.41	10.4	NF	NF	2.77	8.37	76.3
PFOS	86	11.4	10.5	NF	4.16	9.65	15.0	74.6
PFNS	58	1.12	1.86	NF	NF	0.43	1.30	13.7
ΣPFSAs		248	375	NF	73.6	127	277	3925

NF: Below the detection limit.

Table S8. Regression coefficient (β) for ln-transformed urinary creatinine-adjusted PFASs (pg/g-Cre) with ln-transformed daily intake of size-resolved PM_{10}-bound PFASs (pg/day) via inhalation among warehouse workers by linear mixed effect models (LME).

	$PM_{0.43–0.65}$	$PM_{0.65–1.1}$	$PM_{1.1–2.1}$	$PM_{2.1–3.3}$	$PM_{3.3–4.7}$	$PM_{4.7–5.8}$	$PM_{5.8–9.0}$	$PM_{9.0–10}$
PFBA	0.106	-0.083	-0.156	-0.199	-0.037	-0.077	0.112	-0.123
PFPeA	-0.089	-0.005	-0.015	-0.035	-0.041	-0.052	-0.055	0.170*
PFHxA	-0.101	0.117	0.291**	0.202**	0.028	0.160	0.041	0.050
PFHpA	0.104	-0.033	0.448*	-0.302	0.531**	0.019	0.169	0.086
PFOA	-0.075	-0.199	-0.199	-0.121	-0.035	-0.147	-0.016	-0.425
PFNA	0.287*	-0.012	0.134	0.234	-0.082	0.015	0.515**	0.055
PFDA	0.201**	0.110*	0.136**	0.076	0.123*	-0.020	-0.034	0.153**
PFBS	0.134**	-0.282	-0.133	0.065	-0.023	0.170**	0.055	0.040
PFHxS	0.072	0.079	0.102*	-0.150	0.035	0.024	-0.667	0.633**
PFOS	0.193*	-0.073	0.111	0.267**	0.074	-0.041	0.118**	-0.212

Warehouse workers: sorters, stevedores, and other people working in the warehouse for 10 h. LME models with adjustments for sex, age, smoking, BMI, job assignment, duration of work history, blood pressure, blood sugar, and blood lipids. Abbreviations: body mass index, BMI. **: $p < 0.01$. *: $p < 0.05$

Table S9. Regression coefficient (β) for the ln-transformed urinary creatinine-adjusted 8-OHdG and MDA (pg/g-Cre) with the ln-transformed urinary creatinine-adjusted PFASs (pg/g-Cre) among warehouse workers by linear mixed effect models (LME).

	8-OHdG				MDA			
	Slope	95% LCI	95% UCL	P	Slope	95% LCL	95% UCL	P
PFBA	0.093	0.036	0.151	**	0.319	0.195	0.444	**
PFPeA	0.034	-0.007	0.074		0.149	0.063	0.234	**
PFHxA	0.070	0.042	0.098	**	0.108	0.043	0.172	**
PFOA	0.137	0.097	0.175	**	0.042	-0.052	0.136	
PFDA	0.177	0.134	0.220	**	0.157	0.058	0.256	**
PFTrDA	0.219	0.162	0.276	**	0.114	-0.032	0.260	
PFBS	0.069	0.027	0.110	**	0.164	0.074	0.253	**
PFPeS	0.075	0.027	0.124	**	0.224	0.129	0.318	**
PFHxS	0.010	-0.021	0.042		0.041	-0.026	0.108	
PFHpS	0.050	0.003	0.096	*	0.135	-0.125	0.396	
PFOS	0.105	0.073	0.138	**	0.105	0.025	0.184	*
PFNS	0.098	0.034	0.162	**	0.080	0.026	0.134	**

Warehouse workers: sorters, stevedores, and other people working in the warehouse for 10 h. LME models with adjustments for sex, age, smoking, BMI, job assignment, duration of work history, blood pressure, blood sugar, and blood lipids. Abbreviations: confidence interval, CI; 8-hydroxydeoxyguanosine, 8-OHdG; malondialdehyde, MDA; linear mixed-effects models, LME; body mass index, BMI. **: $p < 0.01$. *: $p < 0.05$

Table S10. Regression coefficient (β) for ln-transformed urinary creatinine-adjusted 8-OHdG (pg/g-Cre) with ln-transformed daily intake (pg/day) of size-resolved PM_{10}-bound PFASs via inhalation among warehouse workers by linear mixed effect models (LME).

	8-OHdG							
	$PM_{0.43–0.65}$	$PM_{0.65–1.1}$	$PM_{1.1–2.1}$	$PM_{2.1–3.3}$	$PM_{3.3–4.7}$	$PM_{4.7–5.8}$	$PM_{5.8–9.0}$	$PM_{9.0–10}$
PFBA	0.015	0.013	0.104	0.078	0.147**	0.141**	0.093	0.035
PFPeA	-0.072	0.049**	0.002	0.008	-0.032	-0.017	-0.022	0.019
PFHxA	-0.081	0.052**	0.054**	0.019	-0.008	0.014	0.023	0.020
PFHpA	-0.009	-0.001	-0.048	-0.028	-0.054	-0.025	-0.042	0.032
PFOA	-0.005	0.017	0.006	-0.075	-0.016	-0.055	-0.015	-0.018
PFNA	0.011	0.024	-0.014	0.031	0.011	-0.060	0.012	0.002
PFDA	0.014	-0.006	0.016	-0.013	0.026	-0.019	-0.007	-0.023
PFBS	0.031**	-0.054	-0.041	0.033*	0.084**	0.064**	0.043**	0.080**
PFHxS	0.013	0.026	0.001	0.037*	0.026*	0.041*	0.034	-0.083
PFOS	0.053	0.027	-0.028	-0.040	0.002	0.047**	-0.013	0.007
ΣPFCAs	-0.059	0.031	0.037	-0.022	0.019	0.119*	-0.003	0.035
ΣPFSAs	0.014	-0.010	-0.030	0.005	0.049*	0.056**	0.019	0.097**
ΣPFASs	-0.030	0.027	0.005	-0.015	0.041	0.129**	0.002	0.171**
（C4-C7）ΣPFCAs	-0.060	0.032	0.059	-0.017	0.026	0.139*	0.000	0.080
（C8-C10）ΣPFCAs	-0.015	0.001	0.013	-0.034	-0.004	-0.027	-0.011	0.049
（C4-C7）ΣPFSAs	0.032	-0.046	-0.039	0.028*	0.046**	0.067**	0.050*	0.094**

Warehouse workers: sorters, stevedores, and other people working in the warehouse for 10 h. LME models with adjustments for sex, age, smoking, BMI, job assignment, duration of work history, blood pressure, blood sugar, and blood lipids. Abbreviations: 8-hydroxydeoxyguanosine, 8-OHdG; malondialdehyde, MDA; body mass index, BMI. **: $p < 0.01$. *: $p < 0.05$

Table S11. Regression coefficient (β) for ln-transformed urinary creatinine-adjusted MDA (pg/g-Cre) with ln-transformed daily intake (pg/day) of size-resolved PM_{10}-bound PFASs via inhalation among warehouse workers by linear mixed effect models (LME).

	MDA							
	$PM_{0.43–0.65}$	$PM_{0.65–1.1}$	$PM_{1.1–2.1}$	$PM_{2.1–3.3}$	$PM_{3.3–4.7}$	$PM_{4.7–5.8}$	$PM_{5.8–9.0}$	$PM_{9.0–10}$
PFBA	0.042	-0.199	-0.472	-0.254	0.233	-0.285	-0.214	-0.204
PFPeA	0.094	-0.065	0.007	0.032	0.030	-0.058	0.051	-0.062
PFHxA	0.014	0.003	0.062	0.038	0.038	-0.026	0.136**	-0.027
PFHpA	-0.070	-0.044	-0.143	0.080	-0.076	0.001	0.024	0.070
PFOA	-0.021	0.013	0.067	-0.104	-0.034	-0.063	0.104*	0.125
PFNA	-0.158	-0.093	0.001	-0.194	-0.040	-0.107	0.127**	0.069
PFDA	-0.170	-0.000	-0.092	-0.072	-0.065	-0.176	0.077*	-0.022
PFBS	0.045	-0.049	-0.006	-0.017	-0.054	-0.071	0.056	-0.053
PFHxS	-0.149	-0.043	0.029	-0.040	-0.003	-0.035	0.062	-0.100
PFOS	-0.172	-0.140	-0.088	-0.141	-0.025	-0.082	0.019	-0.110
ΣPFCAs	0.050	-0.143	-0.162	-0.035	0.057	-0.332	0.122	-0.318
ΣPFSAs	-0.047	0.106	-0.102	-0.084	-0.001	-0.025	0.063	-0.067
ΣPFASs	0.007	-0.153	-0.164	-0.071	0.030	-0.253	0.118	-0.170
（C4-C7）ΣPFCAs	0.089	-0.131	-0.218	0.020	0.100	-0.360	0.109	-0.475
（C8-C10）ΣPFCAs	-0.039	-0.041	0.018	-0.093	0.003	-0.028	0.103**	0.008
（C4-C7）ΣPFSAs	0.011	-0.024	0.007	0.008	0.058	-0.042	0.055	-0.060

Warehouse workers: sorters, stevedores, and other people working in the warehouse for 10 h. LME models with adjustments for sex, age, smoking, BMI, job assignment, duration of work history, blood pressure, blood sugar, and blood lipids. Abbreviations: 8-hydroxydeoxyguanosine, 8-OHdG; malondialdehyde, MDA; body mass index, BMI. **: $p < 0.01$. *: $p < 0.05$

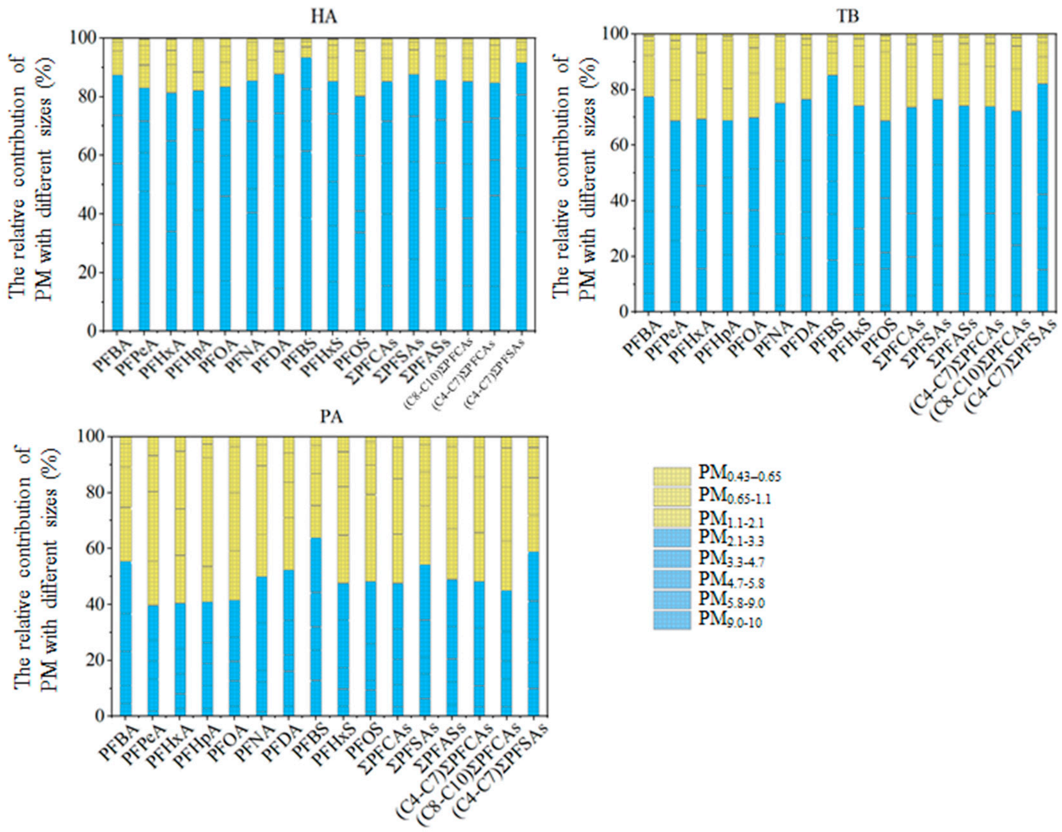

Figure S1. The relative contributions (%) of PM with different sizes to total PFASs deposition in the HA, TB and PA of respiratory tract. Abbreviations: head airway, HA; tracheobronchial, TB; and pulmonary, PA. Un-deposition is the fraction of PFASs not deposited in respiratory tract during respiration. The % is for mass concentrations.

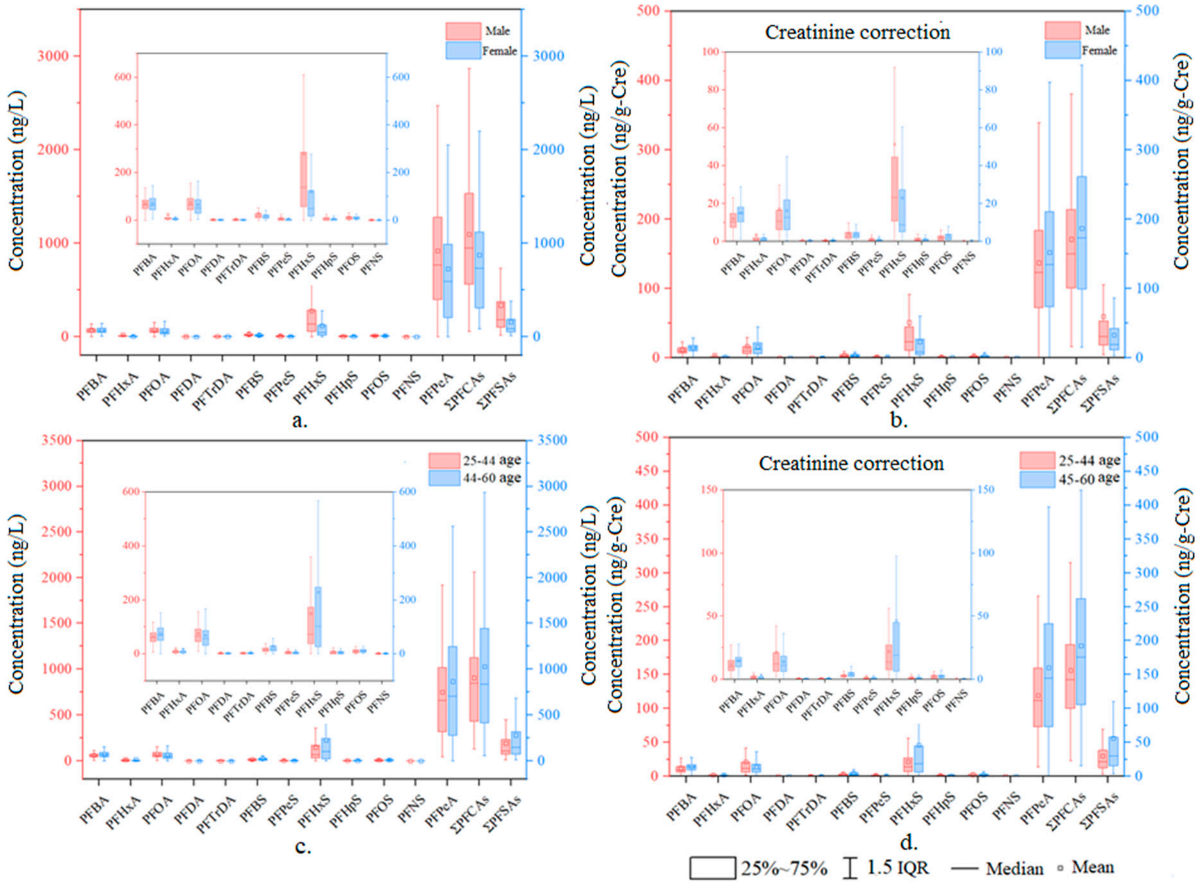

Figure S2. Concentration (μg/L and μg/g-Cre) of PFASs in urine of different gender (a.b) and age groups (c.d) in the waste recycling plant.

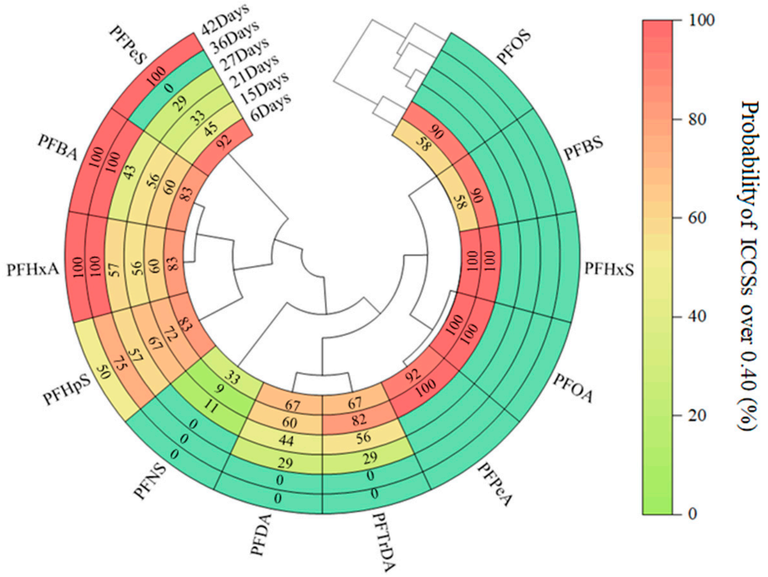

Figure S3. A polar heatmap (%) illustrating the likelihood of intraclass correlation coefficients exceeding 0.40 for ln-transformed concentrations of PFASs adjusted for urinary creatinine, analyzed by sorters across randomly chosen consecutive sampling days through two-way random-effects models.

References

45. USEPA. *Exposure Factors Handbook: 2011 Edition*; National Center for Environmental Assessment, 2011.

71. Pilz J, Meineke I, Gleiter CH. Measurement of free and bound malondialdehyde in plasma by high-performance liquid chromatography as the 2,4-dinitrophenylhydrazine derivative. J Chromatogr B Biomed Sci Appl. 2000, 742, 315–325.
